# Insight into hypoglycemia in pediatric type 1 diabetes mellitus

**DOI:** 10.1186/1687-9856-2012-19

**Published:** 2012-06-20

**Authors:** Kimberly E Lehecka, Venkat S Renukuntla, Rubina A Heptulla

**Affiliations:** 1 Baylor College of Medicine, One Baylor Plaza, Houston, Texas, 77030, USA; 2 Department of Pediatrics, Division of Pediatric Endocrinology, Albert Einstein College of Medicine, 1300 Morris Park Ave, Bronx, NY, 10461, USA; 3 Department of Pediatrics, Division of Pediatric Endocrinology, Albert Einstein College of Medicine/ The Children’s Hospital at Montefiore, 3415 Bainbridge Ave, Bronx, NY, 10467, USA

**Keywords:** Hypoglycemia, Diabetes mellitus, Insulin

## Abstract

Hypoglycemia is a common complication of insulin treatment in type 1 diabetes mellitus and can occur in any patient with diabetes when glucose consumption exceeds supply. Many studies have been done to elucidate those factors that predict severe hypoglycemia: younger age, longer duration of diabetes, lower HgbA_1c_, higher insulin dose, lower Body Mass Index, male gender, Caucasian race, underinsurance or low socioeconomic status, and the presence of psychiatric disorders. Hypoglycemia can affect patients' relationships, occupation, and daily activities such as driving. However, one of the greatest impacts is patients' fear of severe hypoglycemic events, which is a limiting factor in the optimization of glycemic control. Therefore, the importance of clinicians’ ability to identify those patients at greatest risk for hypoglycemic events is two-fold: 1) Patients at greatest risk may be counseled as such and offered newer therapies and monitoring technologies to prevent hypoglycemic events. 2) Patients at lower risk may be reassured and encouraged to improve their glycemic control. Since the risk of long-term complications with poor blood glucose control outweighs the risks of hypoglycemia with good blood glucose control, patients should be encouraged to aim for glucose concentrations in the physiologic range pre- and post-prandially. Advancements in care, including multiple daily injection therapy with analog insulin, continuous subcutaneous insulin infusion, and continuous glucose monitoring, have each subsequently improved glycemic control and decreased the risk of severe hypoglycemia.

## Definition

Hypoglycemia is a common complication of insulin treatment in type 1 diabetes mellitus [1]. A safe blood glucose concentration varies with many factors, including rate of fall of blood glucose, duration of hypoglycemia, and age of the child [2]. Therefore, determining the precise and exact blood glucose that should be considered "low" is difficult. One common benchmark is 65-70 mg/dl (3.6-3.9 mmol/L) because this is the concentration at which counter-regulatory mechanisms are triggered in persons without diabetes [3]. However, symptoms and the clinical significance associated with specific blood glucose concentrations vary with each individual, and it is common for patients with hypoglycemia to be unaware when their glucose is low [4,5]. Young children and infants with type 1 diabetes mellitus (T1DM) represent additional difficulty in recognizing hypoglycemia because they are unable to recognize and also communicate their warning symptoms to their caregivers [6].

Despite the aforementioned difficulties associated with assigning strict categories, the American Diabetes Association's Workgroup on Hypoglycemia has formed a classification system in an attempt to aid communication amongst clinicians and researchers. In general terms, they define hypoglycemia as "an abnormally low plasma glucose concentration that exposes the individual to potential harm." They further define hypoglycemic events as:

a) Severe hypoglycemia. An event in which the patient with diabetes suffers neuroglycopenia to the point of requiring the help of another to administer carbohydrate, glucagon, or other resuscitative measures, sometimes to the point of seizure or loss of consciousness, and including coma or death. Even if plasma glucose level is unrecorded during such an episode, recovery attributed to normalization of plasma glucose concentration is sufficient to qualify the event as severe hypoglycemia.

b) Documented symptomatic hypoglycemia. Measured plasma glucose levels ≤70 mg/dl (3.9 mmol/L) that is associated with typical hypoglycemic symptomatology. Hypoglycemia may present as mood or behavioral changes, especially in young children, or more classically as autonomic symptoms such as hunger, sweating, palpitations, tremor, and confusion.

c) Probable symptomatic hypoglycemia. Episodes when a patient with diabetes reports typical hypoglycemic symptoms in which the blood glucose level is unrecorded

d) Asymptomatic hypoglycemia. Measured plasma glucose levels ≤70 mg/dl (3.9 mmol/L) without typical hypoglycemic symptomatology.

e) Relative hypoglycemia- Episodes of patient with diabetes-reported typical hypoglycemic symptoms associated with measured plasma glucose levels of >70 mg/dl (3.9 mmol/L) [4].

## Etiology

In the simplest of terms, hypoglycemia is the result of glucose consumption in excess of glucose availability [7]. In T1DM, it is primarily the result of supra-therapeutic insulin doses, which lack physiologic feedback inhibition in response to low blood glucose concentration [8]. In addition, hypoglycemic counter-regulatory measures are affected by diabetes. In patients with diabetes, pancreatic α-cells fail to secrete glucagon in response to low blood glucose. This is thought to be due to a signaling defect associated with a lack of endogenous insulin production, since the glucagon response to other stimuli remains intact [9]. Epinephrine, norepinephrine, and cortisol responses to hypoglycemia are also blunted in children and adolescents with well-controlled diabetes [10]. Hypoglycemia-associated autonomic failure syndrome is a description of the "vicious cycle" that results in inadequate epinephrine secretion: recent antecedent episodes of hypoglycemia (as often occur in diabetics on intensive insulin therapy) cause a reduction in the epinephrine response to subsequent hypoglycemic events and lower the glucose level required before the epinephrine response is triggered. Furthermore, patients are often unaware of the fact that they are hypoglycemic and therefore do not take action to reverse this state [11].

Severe hypoglycemia involving loss of consciousness is understood to be due to neuroglycopenia, as brain cells lack the glucose necessary for normal functioning [12]. The mechanism of hypoglycemic seizures is presently unknown. In animal studies, it appears that the Substantia Nigra Pars Reticulata (SNR) is involved in regulating hypoglycemic seizures. Fasting causes both the amount of Gamma-aminobutyric Acid (GABA) and ATP-dependent potassium channels in the SNR to decrease, which may predispose the subject to seizure when severe hypoglycemia is induced [13,14]. In addition, hypoglycemia induces gene expression changes in choline acetyltransferase and acetylcholinesterase gene expression in the cerebellum. The subsequent cerebellar dysfunction may be associated with seizure generation as brain cells lack the glucose they need to function [15].

## Importance

Good glycemic control is essential in reducing the risk and delaying the onset of long-term complications of T1DM such as retinopathy, neuropathy, and nephropathy. However, the Diabetes Control and Complications Trial (DCCT) also showed that strict glycemic control with intensive insulin management is associated with an increased risk of severe hypoglycemic events when compared to conventional treatment [16].

Hypoglycemia can affect patients' relationships, occupation, and daily activities such as driving [17,18]. However, one of the greatest impacts is patients' fear of severe hypoglycemic events, which is a limiting factor in the optimization of glycemic control [19,20]. Therefore, the importance of clinicians’ ability to identify those patients at greatest risk for hypoglycemic events is two-fold:

1) Patients at greatest risk may be counseled as such and offered newer therapies and monitoring technologies to prevent hypoglycemic events.

2) Patients at lower risk may be reassured and encouraged to improve their glycemic control [9].

### Incidence with various treatment modalities

Various regimens are used to treat patients with diabetes, which include:

a) Conventional therapy. Once-daily blood or urine glucose monitoring, one to two daily insulin injections of mixed intermediate and rapid-acting insulin’s, diet and exercise.

b) Intensive insulin therapy or multiple daily injections (MDI). At least four glucose checks daily, three or more insulin injections daily with short-acting insulin before meals; dosages based on planned dietary intake, exercise, and finger-stick blood glucose values [16].

c) Continuous subcutaneous insulin infusion (CSII). An insulin pump is used to deliver a constant basal rate of insulin, with patient-programmed boluses based on dietary intake, exercise, and capillary blood glucose values.

d) Continuous glucose monitoring (CGM). A continuous glucose monitor is used to measure the patient's blood glucose on one to five minute intervals, so the patient may then program their insulin pump to deliver medication accordingly. Alarms may be set to alert the patient when glucose is especially high or low.After the DCCT drew attention to the importance of glycemic control, patients with diabetes were generally transitioned from conventional therapy to MDI, which resulted in reduced hemoglobin A_1c_ (HbA_1c_) values [21]. However, this was associated with a three-fold increase in severe hypoglycemic episodes [22]. Use of analog insulin’s such as Lispro (Humalog) in MDI further improved glucose control without increasing the rate of severe hypoglycemia [23,24].

Studies with a small sample size have demonstrated various effects of switching from MDI to CSII, ranging from slight to moderate improvement in HbA_1c_, and decreasing to increasing rates of hypoglycemia (this variation is especially evident in young children) [25-27]. Findings of these studies are summarized in Table 1. The largely-improved rates of severe hypoglycemic episodes may be partially due to a move from Neutral Protamine Hagedorn Insulin (NPH) to long-acting insulin such as glargine [28].

**Table 1:** Studies comparing multiple daily injections (MDI) vs. continuous subcutaneous insulin infusion (CSII) in pediatric patients

**Author**	**Study Design**	**HbA1c**	**Hypoglycemia**	**Other findings**
**DiMeglio et al**.	- 42 children <5y/o with DM >1 yr were randomly assigned to CSII or MDI	-Improved blood glucose both groups, with better control in CSII group at 3 m, no difference at 6 m follow up	- CSII children had increased meter-detected hypoglycemic events	- High satisfaction rate with CSII
	- HbA1c, severe hypoglycemia, meter-detected hypoglycemia, blood sugar variability, BMI, and satisfaction with therapy were checked at baseline, 3 m & 6 m		- Both groups had one hypoglycemic seizure	
**Litton et al.**	- 9 toddlers with DM and history of severe hypoglycemia and ketoacidosis were treated with MDI for a mean of 13.7 m, followed by CSII for a mean of 12.7 m	- MDI HbA1c average 9.5% ± 0.4%	- MDI: mean 0.52 episodes/month	- Normal growth & development on pump therapy
	-HbA1c, severe hypoglycemia, growth and development, contact with healthcare professionals, and satisfaction with therapy were followed	- CSII HbA1c average 7.9% ± 0.3% (p < 0.001 difference)	- CSII: mean 0.09 episodes/month	- No change in frequency of physician or ER visits for acute hyperglycemia or ketoacidosis
				- >80% decline in contact with healthcare professionals indicating more independence
				- Subjective improvement in quality of life, satisfaction with CSII
**Hanas et al.**	- 1 year cross-sectional study of 7-21 year old patients, with 27 on CSII and 62 on MDI	- MDI HbA1c 8.2% ± 1.6%	*MDI incidence of:*	- No admissions for ketoacidosis in either group during cross-sectional study year
	- HbA1c, severe hypoglycemia, and ketoacidosis were followed, with 5 year follow-up of CSII patients	- CSII HbA1c 8.9% ± 1%, however 67% of patients had high HbA1c as the reason for CSII therapy. Mean baseline 1y before study 9.5%, lowered to 8.9% at 1 and 5 year follow-ups	- Severe hypoglycemia - 40.3/100 patient years	- 5 year follow up of CSII patients revealed incidence of ketoacidosis of 4.7/100 per patient year, indicating low risk
			- With unconsciousness- 12.9/100 patient years	
			- Seizures- 9.7/100 patient years	
			*CSII incidence of:*	
			- Severe hypoglycemia- 11.1/100 patient years	
			- No episodes with unconsciousness or seizure	

In patients with T1DM who are prone to hypoglycemia on MDI, meta-analysis shows the rate of severe hypoglycemia is lessened with CSII, with a rate ratio of 4.19 (2.86 to 6.13). This effect is strongest for patients with the highest frequency of hypoglycemia on MDI, and adult patients who presumably have had a longer duration of diabetes (which is associated with greater risk of hypoglycemia). In addition, HbA_1c_ on CSII is reduced 0.2 - 0.62% when compared to MDI, where patients with the worst blood glucose control on MDI receive the most benefit from CSII [29,30]. However, in another meta-analysis, children were shown to have 0.68 more episodes of minor hypoglycemia perweek on CSII [29]. Continuous glucose monitoring is currently in use to further intensify glucose control by providing real-time feedback that helps patients anticipate and prevent hypo- and hyperglycemic events. In adult patients, use of CSII + CGM improved glycemic control when compared to MDI alone, MDI + CGM, or CSII alone, without an associated increase in severe hypoglycemia [31-34] (Table 1).

### Individual risk factors

In the Diabetes Control and Complications Trial (DCCT), factors that predicted severe hypoglycemia in patients on intensive management included a history of severe hypoglycemia, duration of diabetes, higher baseline HbA_1c_, and lower recent HbA_1c_[35]. Since the DCCT, several studies have found increased incidence of severe hypoglycemia in those patients exhibiting the characteristics listed in Table 2.

**Table 2:** Risk factors for severe hypoglycemia

**Risk Factors for Severe Hypoglycemia**	**Reference number**
Younger age	(30)(18)
Longer duration of diabetes	(30)(18)
Male gender	(30)(18)
Caucasian race	(30)
Lower HbA_1c_	(30)(31)(18)
Higher insulin dose	(18)
Lower BMI	(30)
Lower socioeconomic status or Underinsurance	(30)(31)(18)
Psychiatric disorders (thought to be due to infrequent capillary blood glucose testing and chaotic lifestyle)	(30)

It is important to note that in the study by Allen et al. [36], older age was predictive of adverse outcomes. This is in opposition of the predictive nature of younger age, as found by Rewers et al. [37] and Bulsara et al. [24]. It is likely that Allen et al.'s finding of older age represents the longer duration of diabetes experienced by older subjects, which is corroborated by the other studies, rather than a truly different conclusion.

Patients with previous episodes of severe hypoglycemia have an increased risk for subsequent events [36-38]. Other risk factors associated with recurrent severe hypoglycemia are increased duration of diabetes and underinsurance, at least in adolescents >13 years of age [36]. However, it is important to note that frequent episodes of mild hypoglycemia are not a risk factor for severe hypoglycemia [37,38].

Conversely, a recent study of 108 adolescents showed no significant difference between teenagers who had at least one episode of severe hypoglycemia in the previous year and teenagers without severe hypoglycemia when comparing BMI, HbA_1c_, insulin dose, caloric intake, race, gender, parental education level, parental marital status, and annual family income [39].

### Effect of hypoglycemia on cognition

One of the major theoretical concerns surrounding low blood glucose is the long-term cognitive effects of hypoglycemia to the point of neuroglycopenia. It is known that profound hypoglycemia (blood glucose <18 mg/dL) can cause cerebral energy failure, neuronal necrosis, and cessation of electrical brain activity if maintained for an extended period [40]. However, it is reassuring that prior recurrent episodes of moderate hypoglycemia were found to be protective against neuronal damage and cognitive dysfunction due to profound hypoglycemia in rats [41].

Less severe episodes of hypoglycemia (blood glucose 50-65 mg/dL) are of more concern to the vast majority of patients with diabetes. These events result in transient cognitive defects, which can impact concurrent activities such as driving [18], but these fortuitously fail to show long-term cognitive consequences. Specifically, the DCCT showed that neither treatment regimen (conventional vs. multiple daily injection therapy) nor frequencies of severe hypoglycemia were associated with a cognitive decline over a mean 18 year follow up [42-44]. In meta-analysis, hypoglycemic seizures in children with diabetes conferred inconsistent, clinically insignificant cognitive defects and most current research fails to suggest a detrimental effect of hypoglycemia [45-48].

Individual studies show a variety of neuropsychological effects of diabetes mellitus on children, with early onset of diabetes having the greatest negative influence on cognition [45]*.* Higher HbA_1c_ values, as found in patients on conventional therapy, were associated with moderate declines in psychomotor efficiency (even when patients with stroke, visual impairment, and renal disease were excluded) and motor speed (when the entire study group was analyzed), although this was not evident in adolescent patients [42,43].

### Nocturnal hypoglycemia and post-exercise nocturnal hypoglycemia

Nocturnal hypoglycemia is defined as a blood glucose value < 70 mg/dL after bedtime. Nocturnal hypoglycemia is frequent (twice per month, on average) and often prolonged (81% of episodes were longer than 1 hour in one study) in adults and children with T1DM, especially in those patients who are treating their disease aggressively [49,50]. Hypoglycemia is more likely to occur during the night if a patient has exercised during the preceding afternoon, a phenomenon known as post-exercise nocturnal hypoglycemia [51].

In a study by the Juvenile Diabetes Research Foundation (JDRF) Continuous Glucose Monitoring Study Group, nocturnal hypoglycemia was associated with lower HbA_1c_ values, was most common in patients aged 15-24 years old, and more likely to be prolonged in those patients <25 years old. The same study showed no significant difference in the frequency of nocturnal hypoglycemia when comparing MDI and CSII therapy. On an individual basis, a lower bedtime blood glucose level has been shown to be a significant predictive factor for nocturnal hypoglycemia, while values >130 mg/dl before a bedtime snack correlate with a lower likelihood [51,52].

Various measures have been investigated to determine what may be done to prevent nocturnal hypoglycemia. In a study by Raju et al., a conventional snack, a snack plus acarbose (an α-glucocidase inhibitor), and an uncooked cornstarch bar all delayed the onset of nocturnal hypoglycemia, but did not prevent it from occurring [50]. Administration of the β-adrenergic agonist Terbutaline at bedtime has been shown to prevent nocturnal hypoglycemia, but is associated with the undesirable effect of hyperglycemia [50,53]. In a study by Taplin et al., reduction of basal insulin in patients on insulin pump therapy was shown to be a safe and effective method of raising the blood glucose nadir and preventing post-exercise nocturnal hypoglycemia [53]. The delay between the onset of nocturnal hypoglycemia and the occurrence of a severe hypoglycemic event (seizure) appears to be significant on the order of 2-4 hours, according to one very small report, indicating that continuous glucose monitoring and alarms may help prevent the most dreaded potential side effects of nocturnal hypoglycemia [54].

Traditionally, morning fasting hyperglycemia is attributed to activation of counter-regulatory mechanisms or a fall in insulin levels after an episode of nocturnal hypoglycemia, termed the Somogyi phenomenon. However, the veracity of this phenomenon has long been questioned, and new studies with continuous glucose monitoring have further detracted from this hypothesis [55,56]. One study observed that only 23% of episodes of morning hyperglycemia were consistent with the Somogyi phenomenon (i.e. were associated with nocturnal hypoglycemia), while 77% of episodes were associated with overnight euglycemia or hyperglycemia [56].

## Conclusion

Severe hypoglycemia is an important issue in the care of patients with diabetes, not in small part due to the fear that it imparts in patients and their caregivers. Hypoglycemia can occur in any patient with diabetes when glucose consumption exceeds supply, but certain descriptors are more characteristic of those patients at greatest risk for severe hypoglycemia - younger age, longer duration of diabetes, lower HgbA_1c_, higher insulin dose, lower BMI, male, Caucasian, underinsurance or low socioeconomic status, and the presence of psychiatric disorders[37]. Patients with a history of severe hypoglycemia are at increased risk of having recurrent episodes, but frequent episodes of mild hyperglycemia are not predictive of severe events. Patients must be especially careful at night, particularly after exercising during the day.

Hypoglycemia does result in transient cognitive defects, which can affect concurrent activities, but has not been shown to significantly impact cognition, except at extremely low blood glucose values (<18 mg/dl)[57]. Good glycemic control is essential in reducing the risk and delaying the onset of long-term complications of diabetes such as retinopathy, nephropathy, neuropathy, and neuropsychological effects, including decreased cognition, psychomotor efficiency, and motor speed[1]. The risk of long-term complications with poor blood glucose control outweighs the risks of hypoglycemia with good blood glucose control, and patients should be encouraged to aim for glucose concentrations in the physiologic range pre- and post-prandially. Advancements in care, including multiple daily injection therapy with analog insulin, continuous subcutaneous insulin infusion, and continuous glucose monitoring, have each subsequently improved glycemic control and decreased risk of severe hypoglycemia.

## Abbreviations

T1DM, Type 1 diabetes mellitus; BMI, Body mass index; MDI, Multiple daily injections; CSII, Continuous subcutaneous insulin infusion; SNR, Substantia nigra pars reticulata; GABA, Gamma-aminobutyric acid, DCCT, Diabetes control and complications trial; CGM, Continuous glucose monitoring; NPH, Neutral protamine Hagedorn; JDRF, Juvenile diabetes research foundation.

## Competing interests

All authors do not have any Financial or Non- Financial Competing Interests to disclose.

## Authors’ contributions

**KL** made substantial contributions to conception, involved in drafting the manuscript and revising it critically for important intellectual content. **VR** actively involved in drafting the manuscript and revising it critically for important intellectual content. **RH** made substantial contributions to conception, involved in drafting the manuscript and revising it critically for important intellectual content and also given final approval of the version to be published. All authors read and approved the final manuscript.

## Author’s information

**RH** is the chief of the Division of Pediatric Endocrinology at Albert Einstein College of Medicine/ The Children’s Hospital at Montefiore and is a very well-known Clinical Researcher in the field of type 1 diabetes mellitus.
